# Symmetric dimeric bisbenzimidazoles DBP(n) reduce methylation of *RARB* and *PTEN* while significantly increase methylation of rRNA genes in MCF-7 cancer cells

**DOI:** 10.1371/journal.pone.0189826

**Published:** 2018-01-12

**Authors:** Svetlana V. Kostyuk, Margarita A. Kvasha, Daria A. Khrabrova, Olga V. Kirsanova, Elizaveta S. Ershova, Elena M. Malinovskaya, Natalia N. Veiko, Alexander A. Ivanov, Vasiliy S. Koval, Alexei L. Zhuze, Vadim H. Tashlitsky, Pavel E. Umriukhin, Sergey I. Kutsev, Elizaveta S. Gromova

**Affiliations:** 1 Research Centre for Medical Genetics, Moscow, Russia; 2 Chemistry Department, Moscow State University, Moscow, Russia; 3 N.N. Blokhin Russian Cancer Research Center, Moscow, Russia; 4 Engelhardt Institute of Molecular Biology, Moscow, Russia; 5 I.M. Sechenov First Moscow State Medical University (Sechenov University), Moscow, Russia; 6 P.K. Anokhin Institute of Normal Physiology, Moscow, Russia; Texas A&M University, UNITED STATES

## Abstract

**Background:**

Hypermethylation is observed in the promoter regions of suppressor genes in the tumor cancer cells. Reactivation of these genes by demethylation of their promoters is a prospective strategy of the anticancer therapy. Previous experiments have shown that symmetric dimeric bisbenzimidazoles DBP(n) are able to block DNA methyltransferase activities. It was also found that DBP(n) produces a moderate effect on the activation of total gene expression in HeLa-TI population containing epigenetically repressed avian sarcoma genome.

**Principal findings:**

It is shown that DBP(n) are able to penetrate the cellular membranes and accumulate in breast carcinoma cell MCF-7, mainly in the mitochondria and in the nucleus, excluding the nucleolus. The DBP(n) are non-toxic to the cells and have a weak overall demethylation effect on genomic DNA. DBP(n) demethylate the promoter regions of the tumor suppressor genes *PTEN* and *RARB*. DBP(n) promotes expression of the genes *RARB*, *PTEN*, *CDKN2A*, *RUNX3*, *Apaf-1* and *APC* "silent" in the MCF-7 because of the hypermethylation of their promoter regions. Simultaneously with the demethylation of the DNA in the nucleus a significant increase in the methylation level of rRNA genes in the nucleolus was detected. Increased rDNA methylation correlated with a reduction of the rRNA amount in the cells by 20–30%. It is assumed that during DNA methyltransferase activity inhibition by the DBP(n) in the nucleus, the enzyme is sequestered in the nucleolus and provides additional methylation of the rDNA that are not shielded by DBP(n).

**Conclusions/Significance:**

It is concluded that DBP (n) are able to accumulate in the nucleus (excluding the nucleolus area) and in the mitochondria of cancer cells, reducing mitochondrial potential. The DBP (n) induce the demethylation of a cancer cell’s genome, including the demethylation of the promoters of tumor suppressor genes. DBP (n) significantly increase the methylation of ribosomal RNA genes in the nucleoli. Therefore the further study of these compounds is needed; it could lead to the creation of new anticancer agents.

## Introduction

DNA methylation is a common epigenetic genome modification that plays an important role in the regulation of many cellular processes, including the control of gene expression in eukaryotes. In eukaryotic cells the DNA is methylated by the DNA-methyltransferases (MTases) of the Dnmt family that methylate C5 carbon atom of the cytosine residue in CpG sequences [1, 2]. Distribution of methylated and nonmethylated CpG sequences in the genome generates a methylation profile that is created *de novo* by enzymes Dnmt3a and Dnmt3b in the course of the embryogenesis and is copied each round of the replication by maintenance Dnmt1 [2]. CpG islands in the regulatory regions of the active genes are usually not methylated.

In many cancer tumors hypermethylation of CpG islands is detected in the promoter regions of various genes, including the tumor suppressor genes, cell cycle regulator genes, DNA repair genes, which leads to their silencing [3,4]. However, the hypermethylation of promoters of individual genes is a potentially reversible process. Therefore, a promising new strategy in the cancer therapy was proposed by the reactivation of genes responsible for tumor suppression by the DNA demethylation [5]. It is known that MTases inhibitors can effectively reactivate tumor suppressor genes. Currently several such inhibitors are known [5, 6]. However, all known inhibitors of MTases possess a number of disadvantages, like the instability in aqueous solutions and high cytotoxicity [5], probably due to the nonspecific incorporation of these drugs into the DNA. Therefore the search for new inhibitors that are not embedded in the DNA is extremely important. In particular, compounds that block the contacts of MTases with the DNA can work as such methylation inhibitors.

**Fig 1:**
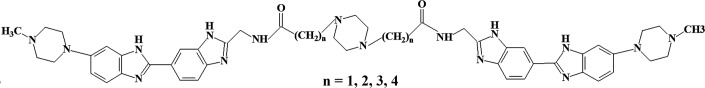
Symmetric dimeric bisbenzimidazoles; bisbenzimidazole fragments joined by oligomethylene linkers with a central 1,4-piperazine residue (DBP(1–4)).

Dimeric bisbenzimidazoles, DB(n), that are produced by two fragments of Hoechst33258 analogue, connected by a linker with a different number (n) of methylene groups were recently synthesized and characterized [7]. The DB(n) contact with the minor groove of the DNA double helix [7] and may inhibit the activity of the catalytic domain of eukaryotic MTase Dnmt3a *in vitro* (IC 50 5–78 μm) [8]. These compounds are not toxic for the cells in a wide concentration range and can penetrate through the cell membranes [9]. However, the poor water solubility of DB(n) limits their application in living systems. Further research included the synthesis of the dimeric bisbenzimidazoles having a 1,4-piperazine cycle in the oligomethylene linker between bisbenzimidazole fragments, DBP(n) (Fig 1). These compounds are water soluble, able to bind the DNA and relatively low toxic [10]. In experiments on the model systems it was shown that the DBP(n) in micromolar concentrations *in vitro* inhibit prokaryotic MTase M.SssI [10]. It was also found that the DBP(n) produces a moderate effect on the activation of total gene expression in HeLa-TI population containing epigenetically repressed avian sarcoma genome [10].

## Materials and methods

### Cell culture

ER/PR-positive MCF-7 breast cancer cells were purchased at ATCC, Manassas, USA (Cat: HTB-22). Ethical approval for the use of human cells was obtained from the Committee for Medical and Health Research Ethics of Research Centre for Medical Genetics, Russian Academy of Medical Sciences (2012, approval number 5). MCF-7 cells were cultured in DMEM medium supplemented with 10% (v/v) fetal calf serum, 2 mM L-glutamine, 100 units/mL penicillin, and 100 μg/mL of streptomycin. Cells were grown in a humidified atmosphere with 5% CO2 in air at 37°C. Before treatment with DBP (1–4) or 5-aza-2′-deoxycytidine (Sigma), cells were grown for 24 h in slide flasks.

### Flow cytomery (FCA)

Before flow cytometry, the cells were washed in Versene solution, then treated with 0.25% trypsin under the control of light microscopic observation. The cells were transferred to the Eppendorf tubes, washed with culture media, then centrifuged and resuspended in PBS. The staining of the cells with Ki-67 or 5mC antibodies was performed as described below. Briefly, to fix the cells, the paraformaldehyde (PFA, Sigma) was added at a final concentration of 2% at 37°C for 10 min. The cells were washed three times with 0.5% BSA-PBS and permeabilized with 0.1% Triton X-100 (Sigma) in PBS for 15 min. Cells (~ 50 x 103) were washed three times with 0.5% BSA-PBS and stained with 1–2 μg/mL FITC- Ki-67 antibody or 5mC antibodies (Abcam) for 3 h at 4°C, then again washed thrice with 0.5% BSA-PBS. In the case of 5mC, cells were stained with 1 μg/mL secondary FITC-conjugated antibodies (Abcam) for 1 h at 4°C. To quantify intracellular DNA, the cells were treated with propidium iodide and RNAase A. To quantify the background fluorescence, we stained a portion of the cells with secondary FITC-conjugated antibodies only. The cells were analyzed at CyFlow Space (Partec, Germany). The results were calculated as the medians of FL parameters and are presented as a mean of medians obtained in three independent experiments.

#### Fluorescent microscopy

Cell images were obtained using the AxioScope A1 microscope (Carl Zeiss). More than 30 photos of cells were made with each filter used.

#### DBP (1–4) fluorescence

The cells were cultivated in the presence of DBP (1–4). They were washed with PBS for 10 min at room temperature and covered with cover glasses. The fluorescence was analyzed with filter 2 (λ ex = 380 nm).

### Immunocytochemistry

MCF-7 cells were fixed in 3% formaldehyde (4°C) for 20 min, washed with PBS and then permeabilized with 0.1% Triton X-100 in PBS for 15 min at room temperature, followed by blocking with 0.5% BSA in PBS for 1 h and incubated overnight at 4°C with the 5mC antibody (Santa Cruz Biotechnology). After washing with 0.01% Triton X-100 in PBS MCF-7 cells were incubated for 2 h at room temperature with the FITC goat anti-sheep IgG, washed with PBS and then stained with DAPI. To quantify the background fluorescence the cells were stained with secondary FITC-conjugated antibodies only. Fluorescence was analyzed using filter 4 (λ ex = 490 nm) for FITC and filter 2 for DAPI.

#### Mitochondria

The cells were stained with 30 nM TMRM (tetramethylrhodamine methyl ester) (Molecular Probes) for 15 min at 37°C and washed with PBS for 10 min at room temperature, after that they were covered with the cover glasses. Fluorescence was analyzed using a filter 6 (λ ex = 520 nm) for TMRM and a filter 2 for DBP(1–4).

### Extraction of DNA

To extract the DNA, the cells were removed from the media by the centrifugation at 460 x g, followed by mixing of 3 mL of the solution containing 1% sodium lauryl sarcosylate, 0.02 M EDTA, and 75 μg/mL RNAse A (Sigma, USA), incubation for 45 min, then the 24-h treatment with proteinase K (200 μg/mL, Promega, USA) at 37°C. The DNA was extracted from lysed cells. After two cycles of the purification with saturated phenolic solution, DNA was precipitated by adding two volumes of ethanol in the presence of 2M ammonium acetate. The precipitate was then washed with 75% ethanol twice, then dried and dissolved in water. The concentration of DNA was determined by measuring fluorescence intensity after DNA staining with the RiboGreen (Molecular Probes/Invitrogen, CA, USA).

### Quantification of mRNA levels

Total mRNA was isolated from the cells using RNeasy Mini kit (Qiagen, Germany). After the treatment with DNAse I, RNA samples were reverse transcribed by Reverse Transcriptase kit (Sileks, Russia). The expression profiles were obtained using quantitative reverse transcriptase polymerase chain reaction (qRT-PCR) with SYBRgreen PCR MasterMix (Applied Biosystems).

The mRNA levels were analyzed in several independent experiments using the StepOne Plus (Applied Biosystems); the technical error (%CV) was approximately 2%. All PCR products were run in the polyacrylamide gel (PAGE) to confirm their size. The following primers were used (Sintol, Russia) (Table 1):

**Table 1:** 

***CDKN2A***	**F: ATGGAGCCTTCGGCTGACT**	**R: GTAACTATTCGGTGCGTTGGG**
***Apaf-1***	**F: GGCCTATTGGGAGAAATCCAC**	**R: ACCACTGCCAAATGGTTTTGT**
***RUNX3***	**F: AGGCAATGACGAGAACTACTCC**	**R: CGAAGGTCGTTGAACCTGG**
***APC***	**F: CCTCATCCAGCTTTTACATGGC**	**R: GCCCGAGCCTCTTTACTGC**
***RARB***	**F: CGTGGAGTTTGCTAAACGTCT**	**R: TGGTGTCTTGTTCTGGGGTAT**
***PTEN***	**F:TTTGAAGACCATAACCCACCAC**	**R: ATTACACCAGTTCGTCCCTTTC**
***TBP***	**F: GCCCGAAACGCCGAATAT**	**R: CCGTGGTTCGTGGCTCTCT**
***18SrRNA***	**F:CTGAGAAACGGCTACCAC**	**R: TGGTGCCCTTCCGTCAAT**
***5-ETS (1)***	**F: AACGGTGGTGTGTCGTTC**	**R: TCTCGTCTCGTCTCACTCAA**
***5-ETS(2)***	**F: GCAGGTGTTTCCTCGTACC**	**R: TCAGATCGCTAGAGAAGGCT**
***3-ETS***	**F: CCCGCTTCTTCGGTTCC**	**R: GGCTCCCAAACCACGCT**

Two independent experiments were performed. The amounts of RNA in each experiment were determined in three parallel samples.

### DNA methylation analysis

The effects of DBP(1–4) on promoter methylation of the selected genes (rDNA, *PTEN* and *RARB*) were estimated using methylation-sensitive restriction analysis (MSRA). 100 ng of DNA were digested with HpaII or MspI in 20 μL of «Tango» buffer (1X Buffer composition: 33 mM Tris-acetate (pH 7.9 at 37°C), 10 mM magnesium acetate, 66 mM potassium acetate, 0.1 mg/ml BSA) according to the manufacturer’s instruction (18 h, 37°C). The DNA was precipitated by adding two volumes of ethanol in the presence of 3M sodium acetate. The precipitate was then washed with 75% ethanol twice, then dried and dissolved in water.

Quantitative real-time PCR analysis of DNA was performed on the StepOnePlusTM Real-Time PCR System (“Applied Biosystems”) using the SYBR Green dye, analyzing four genomic sequences: ribosomal repeat rDNA (A (5-ETS, 541–651 b) and B (18S rRNA, 4894–4987 b), genes *ACTB*, *RARB* and *PTEN*. In order to construct the calibrating dependence we employed the reference standard sample of DNA used to prepare 9 dilutions containing from 108 to 50 copies of DNA in 1 ml. The dependence of the threshold cycle value from the DNA amount in the samples tested was linear within the range of concentrations varying from 50 to 108 copies, with efficacy of amplification (E) amounting to 96.8%. Average values for three measurements and SD are shown. The following primers were used (Table 2):

**Table 2:** 

***ACTB***	**F: GCGGGAAATCGTGCGTGACATT**	**R: GATGGAGTTGAAGGTAGTTTCGTG**
***PTEN***	**F: CAGCCGTTCGGAGGATTATTC**	**R: GGGCTTCTTCTGCAGGATGG**
***RARB***	**F: CTCGCTGCCTGCCTCTCTGG**	**R: GCGTTCTCGGCATCCCAGTC**
***rDNA***	**F: GAACGGTGGTGTGTCGTTC**	**R: GCGTCTCGTCTCGTCTCACT**
**rDNA**	**F: GCCCGAAGCGTTTACTTTG**	**R: CCGCGGTCCTATTCCATTAT**

The methylation index for rDNA is equal to the ratio of rDNA fragment content in HpaII-hydrolyzate and MspI-hydrolyzate. The methylation index for genes *ACTB*, *RARB* and *PTEN* is equal to the ratio of the content of the gene fragment in the HpaII-hydrolyzate and intact DNA (iDNA). The amount of rDNA in the genome MCF-7 was determined with the respect to the internal standard (*ACTB*). All results were confirmed in three independent experiments.

### ROS assay

The cells were analyzed using total fluorescence assay in the 96-well plate format at λ_ex_ = 488 nm and λ_em_ = 528 nm (EnSpire equipment, Finland). MCF-7 were treated with 5 μM H_2_DCFH-DA (Molecular Probes/Invitrogen, CA, USA) for 1–30 min at 37°C.

16 repeated measurements were provided for each DBP(n) concentration and 24 for control. The mean absolute intensities were divided by the average value of the intensity corresponding to t = 0, obtaining the values of I_ROS_. The graphs are presented in the coordinates I_ROS_−time (t). The obtained data were approximated by linear dependence; the value of the tangent of the slope together with the error of determination (index k) was calculated. The obtained index k values were averaged for control and each DBP(n). The dependence of the index Δk/k_**0**_ on the concentration of DBP(n) is demonstrated, k_**0**_ – reaction rate constant for DCF formation in control cells; Δ k = k_**DBP**_−k_0._

### Analysis of 5mC with UPLC/MS/MS

1 μg of the genomic DNA was digested with 2 u of nuclease P1 in 50 μL for 14 h. Then DNA was digested with alkaline phosphatase (4 u) in 50 μL for 4 h. The resulting mixture of nucleosides was analyzed by UPLC/MS/MS. A system consisting of an Acquity liquid chromatograph and a tandem quadrupole MS detector TQD was used. 11.2 μl of diluted sample solutions on an Acquity HSS T3 (1.8 μm) column at 35°C and a flow rate of 0.5 ml/min were used. Eluent was 5% acetonitrile in water in a mixture with 10 mM ammonium acetate (pH 7.0). The quantitative content of nucleosides in the samples was determined from the values of the areas under the peaks on the MRM chromatograms of individual ions. The calibration dependence, constructed for this device and a given set of instrument parameters, was used. As an internal standard, 5-(methyl-d3) -2'-deoxycytidine was used. The percentage of 5mC according to the MS data was estimated by normalizing the amount of m5dC by the amount of dA in the same sample. dA was used for normalization due to the intensity and selectivity of the response for a given nucleoside. Three independent experiments were carried out. The average contents of 5mC were determined for 2 samples of DNA. The data are presented as mean values for three independent experiments.

### Analysis of 5mC content with IFA

Nitrocellulose «Optitran BA-S 85» was incubated in 20 X SSC buffer, dried for 16 hours. To 10 μl of the DNA sample, 1.5 μl of 1 M NaOH was added, incubated for 10 minutes and neutralized with 8.5 μl of a solution of ammonium acetate (4M). 3–5 spots of 1.5 μl of the sample were applied to a filter. The filter was heated for 1.5 hours at 80°C and wetted in a solution of SSC. Prevention of nonspecific sorption of the antibodies was carried out by blocking the membrane with 0.5% BSA solution, 0.1% milk in PBS for 1 hour at 37°C. The filters were incubated in a solution of specific antibodies to 5mC in PBS at a dilution of 1: 250 for 2 hours at 37°C, washed with a 0.05% TWEEN-20 in PBS and incubated at 37°C for 1h in a solution of secondary antibodies conjugated with horseradish peroxidase at a dilution of 1:5000 in PBS containing 0.5% BSA. The filter was washed with a solution of PBS with 0.05% TWEEN-20 and poured into the prepared TMB (for membrane, Sigma) solution in the dark. After the precipitate formed, the filter was washed with water. The intensity of the stained spots was determined by computer analysis of the image of the filter using the "Images6" soft. Three independent experiments were carried out. The average concentrations of 5mC in 3–5 spots of the DNA samples were determined. The data are presented as mean values for three independent experiments.

### Statistics

All reported results were reproduced at least two times as independent biological replicates.

The significance of the observed differences was analyzed using non-parametric Mann-Whitney U-tests. The data were analyzed with StatPlus2007 Professional software (http://www.analystsoft.com/). All p-values were two-sided and considered statistically significant at p < 0.05. The software for 'Imager 6' was designed by R.Veiko (RCMG, Moscow).

## Results

Four DBP(1–4) compounds were previously synthesized [10] (Fig 1). To study the effects of DBP(1–4) on the oestrogen and progesterone receptor positive breast carcinoma cell MCF-7 sterile aqueous solutions of the compounds were added to the cultivation medium at a concentration of 7 to 80 μM and the cells were cultivated for 3 to 72 hours.

The main objective of the study was the analysis of DBP(1–4) influence on the methylation of MCF-7 cells genome. Therefore, the penetration of DBP(1–4) into the cells was examined first and the conditions (concentration and time) in which the proliferative activity of the cells remains stable was determined.

DBP(n) properties are similar to the properties of the dimeric bisbenzimidazole DB(n) containing aliphatic chain in the linker, but DBP(n) have a much better solubility in water. The fluorescent properties of the dimeric bisbenzimidazoles change significantly when the hydrophobicity surrounding the fluorophore is modified [11]. The addition of DBP(n) to DNA resulted in a shift of the emission band maximum from 468 to 475 nm and an increase in the fluorescence intensity [10]. The fluorescence properties of DBP in the hydrophobic environment with DNA were used to analyze the distribution of these compounds in the cells.

### Localization of DBP(1–4) in MCF-7

**Fig 2:**
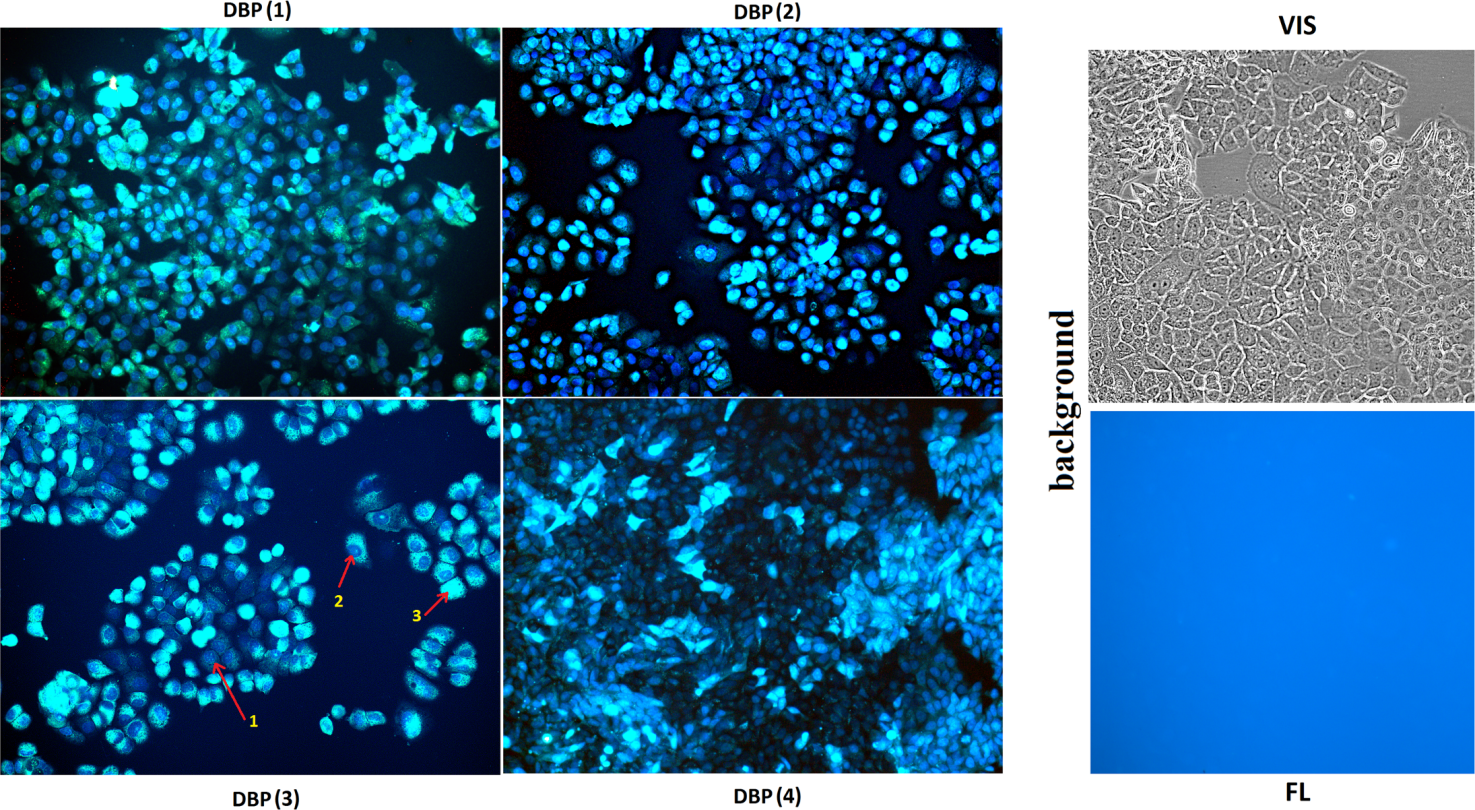
Incorporation of DBP(1–4) in MCF-7 cells. Excitation of fluorescence at 380 nm. Magnification X 20. MCF-7 were incubated with DBP(1–4) (7, 10 or 20 μM) for 24 h and the fluorescence was analyzed in unfixed cells. Staining of the cells was heterogeneous and practically did not depend on the concentrations of DBP(1–4). The 20 μM concentrations of DBP(1–4) were used for an example. The photos of cells were taken with identical exposure. For example the most common nuclei staining types are shown with arrows and numbers: 1 –nucleus is stained only, 2 –nucleus and the cytoplasm are stained. 3- cells show strong blue fluorescence signal while nuclei do not contrast. Background: the photo of control cells in visible light (VIS) and at λex = 380 nm (FL), the exposure is increased two times.

MCF-7 were incubated with DBP(1–4) (7, 10 or 20 μM) for 24 h and then the fluorescence of compounds in unfixed cells was analyzed (Fig 2). It is shown that DBP(1–4) penetrates and accumulates in MCF-7 cells. Staining of the cells was heterogeneous and practically did not depend on the concentrations of DBP(1–4) used. Three main types of staining may be distinguished: (1) the nuclei are colored dark blue; (2) the nuclei are stained dark blue and the cytoplasm in blue color; (3) the cells strongly fluoresce with blue color, the nuclei do not contrast. Thus, DBP(1–4) accumulates in different amounts in the cytoplasm and enters the nucleus.

**Fig 3:**
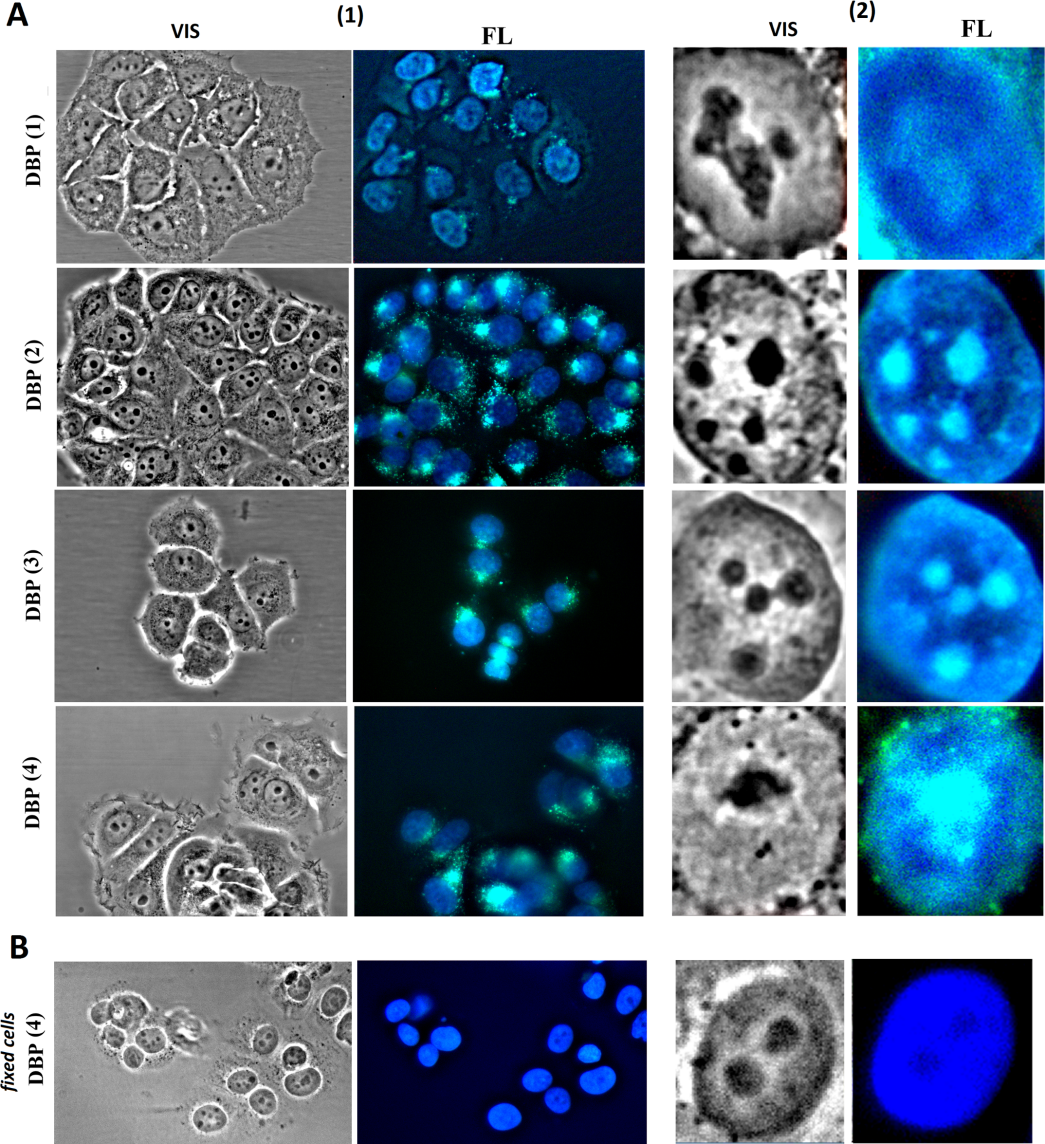
(A) DBP(1–4) localization in MCF-7 cells (1) and cell nuclei only (2). VIS—the photo of control cells in visible light; FL—λex = 380 nm. (1) Magnification X60. (2) For nuclei analysis the image size was increased by an order by computer processing. (B) MCF-7 were fixed with 3% PFA and treated with 0.1% triton X100 solution.

DBP(1–4) are unevenly distributed in the nuclei of MCF-7 (Fig 3A). DBP(1–4) interact with the DNA in the volume of the nucleus, as shown by the dark blue fluorescence which indicates DBP(1–4) complexes with DNA [10]. The compounds also accumulate in close proximity to the nucleoli. The nucleoli are highlighted on the pictures of the cells in a visible light in the form of dense dark spots. Coloring with silver nitrate confirmed that dark spot in the center of the nuclei are nucleoli (the data not shown). In close proximity to nucleoli the wavelength of DBP(1–4) fluorescence is increased, the color changes from dark blue to blue. In the case of DBP(1) the nucleolus contrasts to a much lesser extent, indicating the weaker accumulation of the compounds in these areas. In cells fixed and treated with 0.1% Triton X-100 solution, enhancement of the nucleolus color with the formation of a bright blue color has not been observed (Fig 3B). On the contrary, in the place of a nucleolus, detectable using a visible light microscopy, dark spots were found that indicate the absence of significant quantities of DBP (1–4) in the nucleolus.

**Fig 4:**
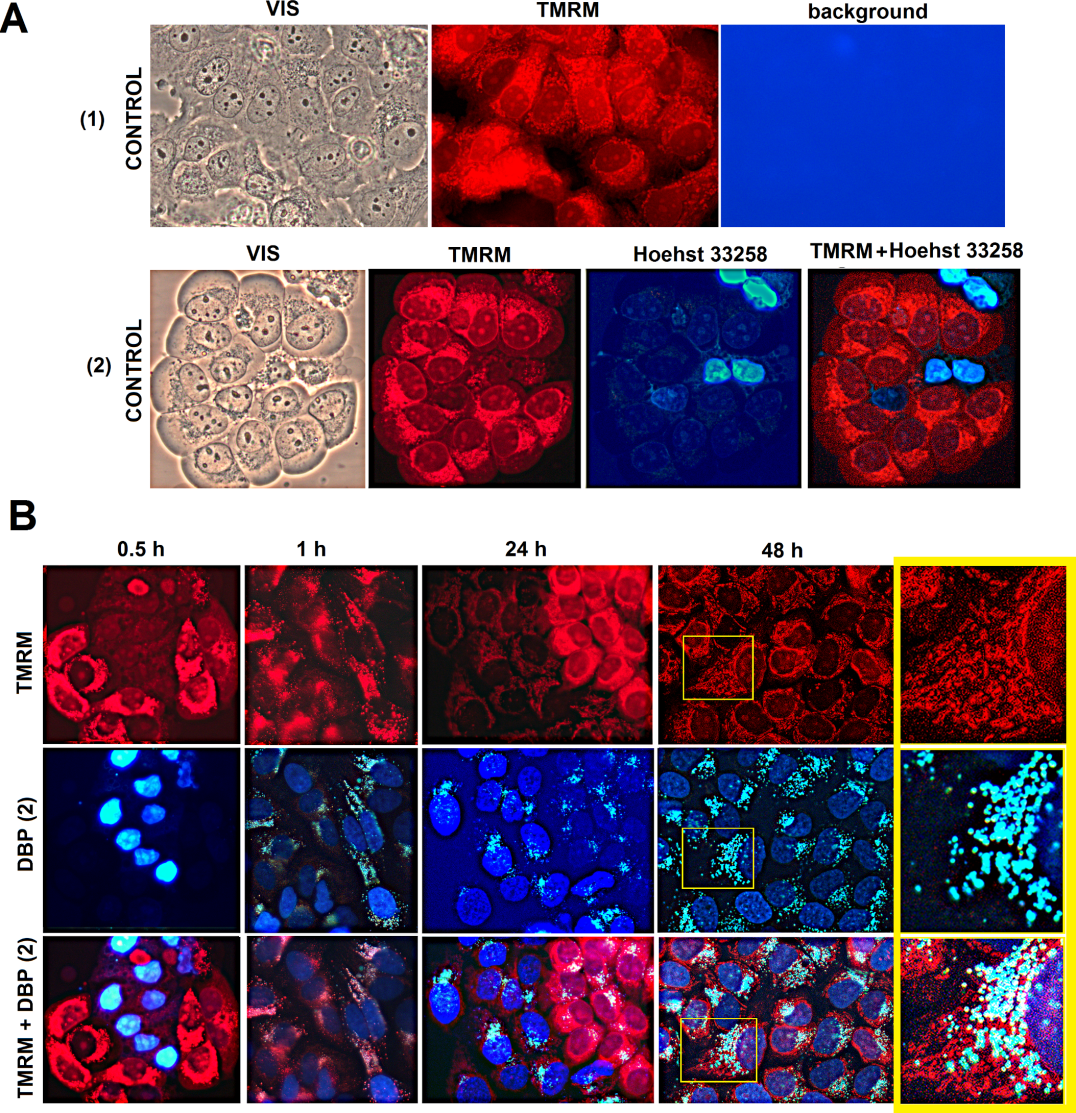
The influence of DBP(1–4) on the MCF-7mitochondria. A. (1)—control cells (stained with MitoTracker TMRM) in visible light (VIS), in λex = 520 nm (TMRM) and in λex = 380 nm (background). (2)—control cells stained with MitoTracker TMRM and Hoechst 33258 (10μM, 0.5 h). Specially selected field shows single cells with a weak mitochondria staining. Particularly these cells accumulate Hoechst 33258 in the nuclei. B. MCF-7 cells treated with 20μM DBP(2) for 0.5–48 h after incubation with MitoTracker TMRM (15 min). For 0.5 h time point we specially selected a field that shows cells with a weak mitochondria staining. DBP(2) quickly penetrates into these cells, similar to Hoechst 33258. Magnification X 60. The cell in a yellow square is enlarged several times for demonstration.

Analysis of DBP(1–4) location in the cytoplasm of unfixed cells suggests that these compounds are localized near/into mitochondria (Fig 3A(1, FL)). To test this hypothesis a known marker for the mitochondria–tetramethylrhodamine methyl ester (TMRM) was used. TMRM interacts with the mitochondrial DNA and is sensitive to the mitochondria potential (Fig 4A(1)). The brighter is the color of the cells, the higher is mitochondrial potential. The cells with damaged mitochondria are colored slightly. The control MCF-7 cells always contain about 3–5% of cells with the signs of the apoptosis: the nuclei have an irregular shape and the mitochondria are slightly colored by TMRM. To identify apoptotic nuclei the dye Hoechst 33258 is usually used, which only penetrates the nuclei of the cells with damaged membrane. Fig 4A(2) shows an example of control cells colored by TMRM and Hoechst 33258 simultaneously. Cells were cultivated with 20μM DBP(1–4) for 0.5, 1, 24 or 48h. Then MitoTracker was added and they were cultivated for another 15 minutes.

The most typical distribution of DBP(1–4) is shown in Fig 4 for DBP(2) as an example. After a short incubation time of MCF-7 with DBP(1–4) (0.5 hours) these compounds do not penetrate into living cells with active mitochondria. In the cells with low mitochondrial potential only the nuclei are stained with DBP(1–4). The nuclei have an irregular shape, which together with the low mitochondria potential indicates the staining of apoptotic or necrotic nuclei of cells culture.

By incubation for one hour DBP(1–4) penetrate into the cells. The nuclei are stained weakly, the nucleolus does not contrast. DBP(1–4) granules accumulate in the cytoplasm, which co-localize with the mitochondria (indicated by pink color on the photo TMRM + DBP(2), Fig 4B). The division of the cells into two subpopulations was observed 24 hours after. The cells with low mitochondrial potential (TMRM weak color) have intensively stained nuclei, i.e., DBP penetrates and accumulates in the nuclei. The cells with a high potential contain DBP(1–4) primarily in the mitochondria, the nuclei are painted poorly. 48 hours after the majority of MCF-7 cells accumulate DBP(1–4) in the nucleus and the cytoplasm. Mitochondrial potential is significantly reduced. The signals from the DBP(1–4) do not coincide with the signals from TMRM. In areas of DBP accumulation in the mitochondria MitoTracker color is reduced most intensively.

### Influence of DBP (1–4) on the level of ROS in MCF-7

**Fig 5:**
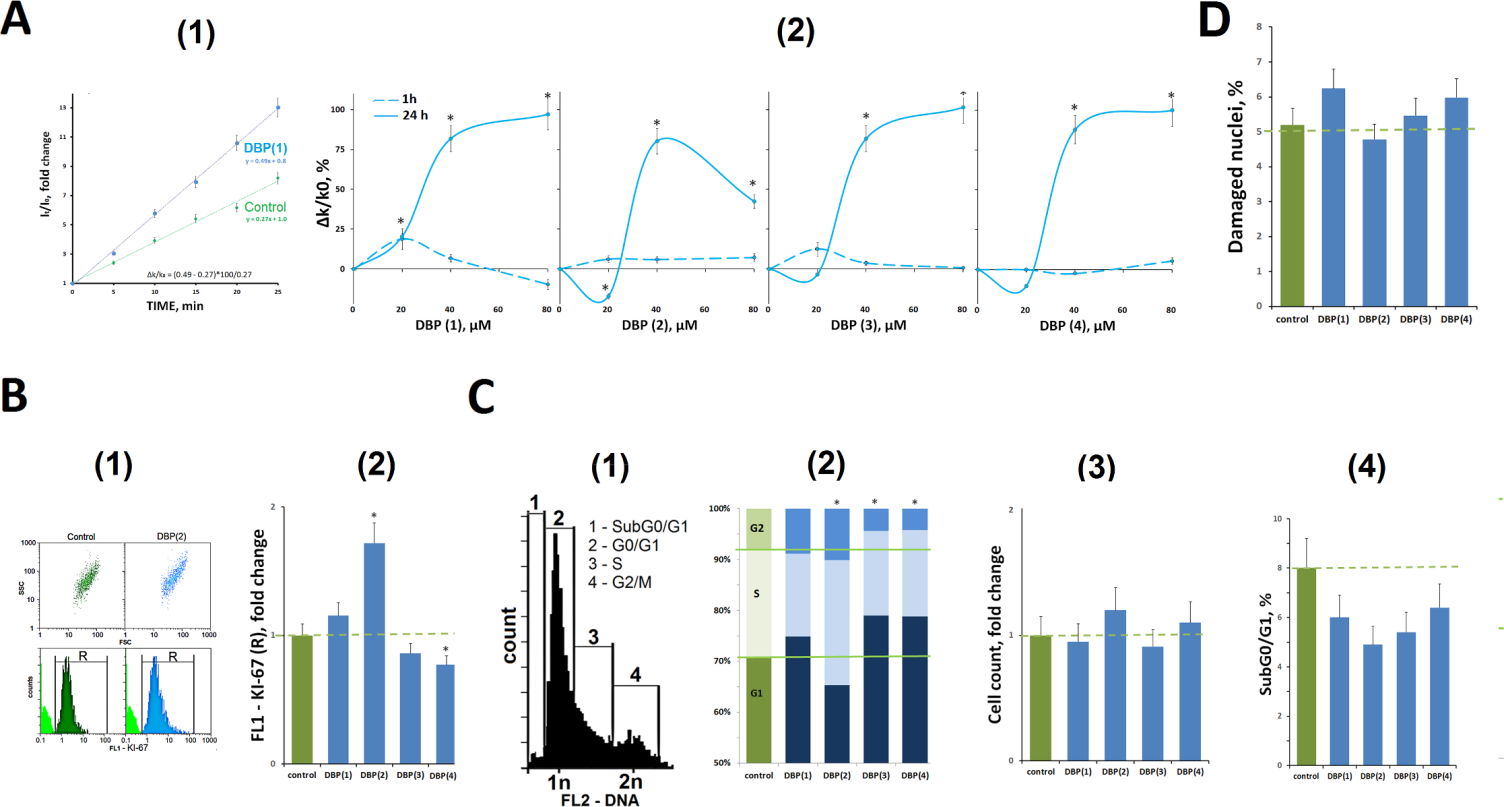
A (FL-reader). (1)–Example of reaction rate constant determination for DCF formation when DCFH reacts with ROS. Cells were cultivated for 24 hours in the presence of 40 μM DBP(1). The cultivation environment was replaced by 5 μm H2DCFH-DA in PBS solution and a relative fluorescence intensity increase was detected. I_t_,I0 –sample’s signal at time t and immediately after H2DCFH-DA addition respectively. The slope of the line– reaction rate constant for DCF formation (k). ROS index Δk/k_0_ = (k_DBP_−k_0_)×100/k_0_ (%). (2)—Relationship between ROS index Δk/k_0_ and DBP(1–4) concentration. Time of cultivation is shown on the figure. B (FCA). Proliferation of MCF-7 cells exposed to DBP(1–4) at final concentration 20 μM for 24 hours. (1)—Distribution of fluorescence of fixed cells stained with anti-Ki-67 antibodies (control—dark green color, DBP(2) as example—blue color). Background fluorescence was quantified using FITC-conjugated secondary antibodies (green color). (2)—The median signal intensity of FL1 (Ki-67+) (gate R). Each experiment was repeated at least three times. Results represent the average of the medians of the FL parameter in independent experiments. C (FCA). (1)—Distribution of fluorescence of fixed cells stained with propidium iodide. The fractions of (1–4) cells with different DNA amounts are shown. (2)–Proportion MCF-7 culture cells with DNA amount corresponding to the G1-, S—and G2/M phases of the cell cycle. The data shown is an average value from three independent experiments. (3) The total number of cells in culture. (4) The number hypodiploid cells (fraction SubG0/G1).D. The proportion of cells in culture with signs of apoptotic nuclei (condensed chromatin, the irregular nucleus shape, see Fig 4). B, C, D–cells were cultivated in the presence of 20 μM DBP(1–4) for 24 hours.

DBP(1–4) localization in the mitochondria suggests that DBP(1–4) may influence the level of ROS. ROS level in cells was measured with known reagent DCHF-DA [12], Fig 5A. Reaction rate constants of DCF formation during the interaction of ROS with cellular DCFH [13] were determined. Within an hour of MCF-7 incubation in the presence of 20–80 μM DBP(1–4) the level of ROS in cells was practically unchanged. 24 hours after an increase in the level of ROS at high concentrations of DBP(1–4) is observed. 20 μM DBP(1–4) concentration has a little effect on the ROS level. Thus, the decrease of MCF-7 mitochondrial potential in the presence of 20 μM DBP(1–4) is not associated with a significant change of ROS level in cells.

### Influence of DBP (1–4) on cell cycle and MCF-7 proliferation

The data obtained by the FCA is shown in Fig 5B. It reflects the impact of DBP(1–4) (20 μm, 24 h) on the number of proliferating cells in the culture. The antibodies to a well-known proliferation marker—protein KI-67 were used. DBP(1,2) stimulated the proliferative activity of the cells MCF-7, while DBP(3,4) on contrary, reduced the number of proliferating cells. A difference was significant only for DBP(2) and DBP(4) (p < 0.05).

To assess the DBP(1–4) impact on cell cycle fixed cells MCF-7 were stained with propidium iodide (Fig 5C(1)). The numbers of the cells in SubG0/G1, G0/G1, S and G2/M phases of the cell cycle were analyzed as described previously [14]. DBP(2) increased the percentage of the cells in S and G2/M phases (p < 0.05). DBP(3,4), on contrary, decreased the content of dividing cells and increased the number of the cells in the phase G0/G1(p < 0.05). However, all these effects were not significant (Fig 5C(2)).

DBP(1–4) produce almost no effect on the total number of the cells in the culture (Fig 5C(3)) and on the number of hypodiploid cells (Fig 5C(4)). The analysis of the cells colored by DBP(1–4) and TMRM (Fig 4B) also shows that the number of apoptotic cells with condensed chromatin and irregular nuclei shape is not influenced by DBP(1–4) (20 μm, 24 h).

### Influence of DBP (1–4) on general DNA methylation in MCF-7 cells

To assess the DBP(1–4) effect on general genomic DNA methylation level three different methods were used: enzyme-linked immunosorbent assay, ultra-high performance liquid chromatography with tandem quadrupole mass spectrometry detection (UPLC/MS/MS) and immunofluorescence analysis.

#### Enzyme-linked immunosorbent assay

**Fig 6:**
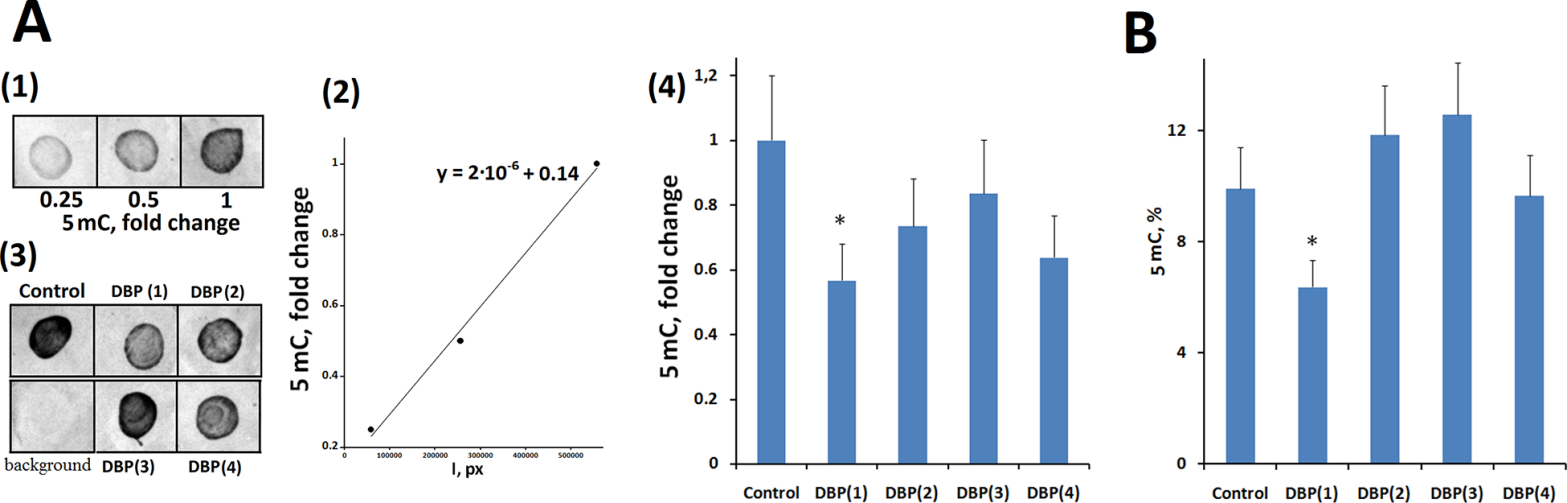
Overall 5-methylcytosine level in DNA of cells treated with 20 μM DBP(1–4) for 72 hours. A (ELISA). (1, 2)–example of calibration dependence between methylated DNA in samples and integral stain density (I). (3) example of DNA samples analysis from cells treated with DBP(1–4). 10 ng of DNA was applied. pBR322 plasmid was used as a negative control. (4) Relative signal change reflecting difference in DNA methylation level. B (*UPLC/MS/MS*). Average 5mC content in DNA samples.

MCF7 cells were incubated with 20 μM DBP(1–4) for 3 days and genomic DNA samples were isolated. Denatured DNA was applied to nitrocellulose filter. The methylation was detected using the antibodies to 5-methylcytosine and secondary antibodies conjugated with horseradish peroxidase. Fig 6A(3) demonstrates the filter with visualized methylated DNA samples. The relative amount of 5-methylcytosine was determined using a calibration dependence of integrated signal intensity and the amount of methylated DNA in the sample (Fig 6A(1,2)). The highest concentration of 5-methylcytosine residues was observed in the DNA isolated from control DBP(1–4) untreated cells (Fig 6A(4)). The concentration of 5-methylcytosine residues in the samples incubated with DBP(1–4) was slightly lower, suggesting a small demethylation effect of each DBP(1–4) compound. Only for the DNA of DBP(1) treated cells the difference with the control was significant.

#### UPLC/MS/MS

Genomic DNA samples isolated from the cells after incubation with DBP(1–4) were hydrolyzed to the nucleosides and the content of 5mC was determined using UPLC/MS/MS. Percentage of the DNA released from the cells is shown in Fig 6B. DBP(2–4) did not affect DNA methylation, but the DBP(1) significantly decreased the DNA methylation (p < 0.05).

#### Immunofluorescence analysis

**Fig 7:**
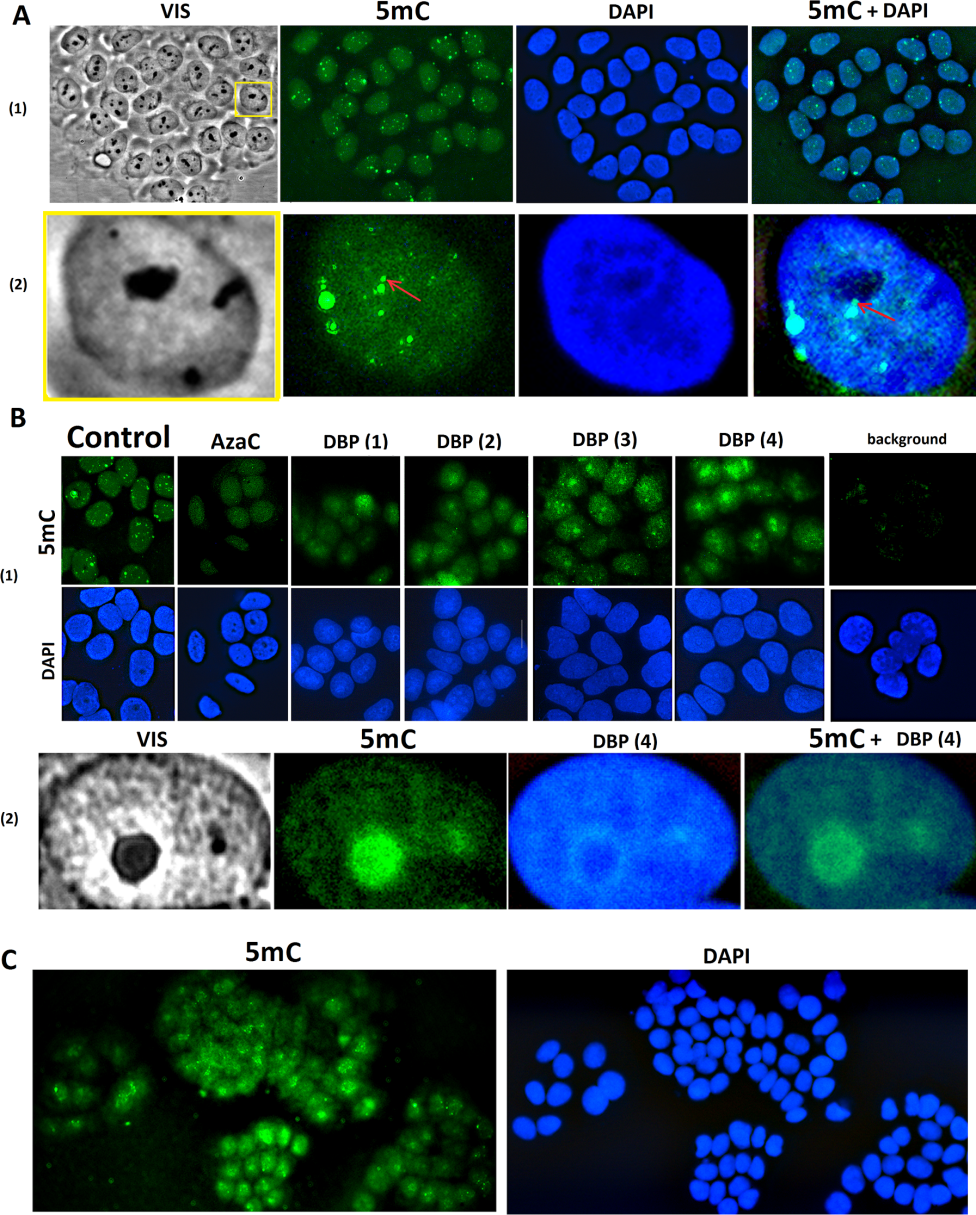
DNA methylation in nuclei of the control MCF-7 cells. A. Cells were processed for immunofluorescence staining with anti 5-mC antibody. (1)—Magnification X60; (2)—For analysis of the nuclei the photo was enlarged by an order using computer processing. The arrow points to the methylated DNA block close to the nucleolus. B. (1)—DNA methylation in nuclei of the control MCF-7 cells and the cells treated with 10 μM AzaC or 20μM DBP(1–4), 72 h. Background: Control cells were only treated with secondary antibodies conjugated with FITC. (2) The photo was enlarged considerably for the analysis of nuclei size. The nucleus of the cell cultivated with DBP(4) is shown for an example. C. An example of the different DNA methylation level in cells’ nuclei that were cultivated in the presence of 20μM DBP(4), 72 h. A,B(1) and C—Cell nuclei were additionally colored with DAPI.

Primary 5-methylcytosine antibodies and secondary antibodies conjugated with FITC were used for immunofluorescence analysis. The localization of 5-methylcytosine residues in the nuclei of control MCF-7 is shown in Fig 7A(1,2). On the background of even nuclei staining regions of the nuclei with hypermethylated DNA sequences were clearly detected. The nucleoli analysis in the nuclei showed that small hypermethylated DNA block is typically localized next to one of the nucleoli Fig 7A(2).

In the presence of 20μM DBP (1–4) (72 h) staining varies considerably (Fig 7B). Color intensity in the nucleus decreases, the spots become more blurred and most of the signals are shifted toward the center of the nucleus. The most significant changes were found in the nuclei of DBP(4) treated cells. Fig 7B(2) shows data for DBP(4) as example. The nucleolus contrasts in cells photographed in a visible light (Fig 7B(2), VIS). In fixed cells DBP(4) does not stain the area of the nucleolus (the dark spots on the background of dark blue staining, Fig 3B, Fig 7B). Obviously the signal from hypermethylated sequences is localized in the nucleoli (Fig 7B(2)). In the presence of 10 μM AzaC accumulation of methylated DNA in the nucleolus was not observed. The methylcytosine concentration was reduced throughout the nuclei (Fig 7B(1)).

Another feature of 5-methycytosine stained nuclei was found. Fig 7C shows a photo of five clones of MCF-7. It is obvious that the staining in different clones varies significantly. Thus, the demethylation of DNA in the cell culture is heterogeneous. However, all clones exhibit the same pattern of methylation change compared with control since the label is concentrated in the nucleolus region.

### The influence of DBP (1–4) on the methylation of individual genes

The cells were cultured in 20 μM DBP(1–4) for 3 days. To analyze the changes in methylation of specific genome sequences the technology of DNA digestion with methylation- sensitive (HpaII) or insensitive (Msp1) restriction endonucleases was used. To confirm the completeness of the hydrolysis of DNA with Msp1 and HpaII endonuclease the content of standard *ACTB* gene was also analyzed. Analyzed fragment of *ACTB* gene contains one demethylated site HpaII/Msp1. After the DNA hydrolysis in the HpaII/Msp1–hydrolysates the *ACTB* gene fragment is not detected.

#### The ribosomal genes

The content of external transcribed spacer fragment A 130 base pairs (5-ETS rDNA 541–670 b) and the fragment B 93 base pairs (18S rRNA, 4894–4987 b) in MCF-7 genome was analyzed. The ACTB gene was used as internal standard. As it was shown by qPCR MCF-7 contains 422 copies of the ribosomal repeats. The fragment A contains 4 HpaII/Msp1 CCGG sites. The fragment B contains one HpaII/Msp1 CCGG site. Quantitative analysis of the rDNA in HpaII-hydrolysate detects the methylation of the A and B fragments. About 15% of rDNA copies contain A fragment with all four CCGG sites methylated. About 12% of rDNA copies contain B fragment with CCGG site methylated.

**Fig 8:**
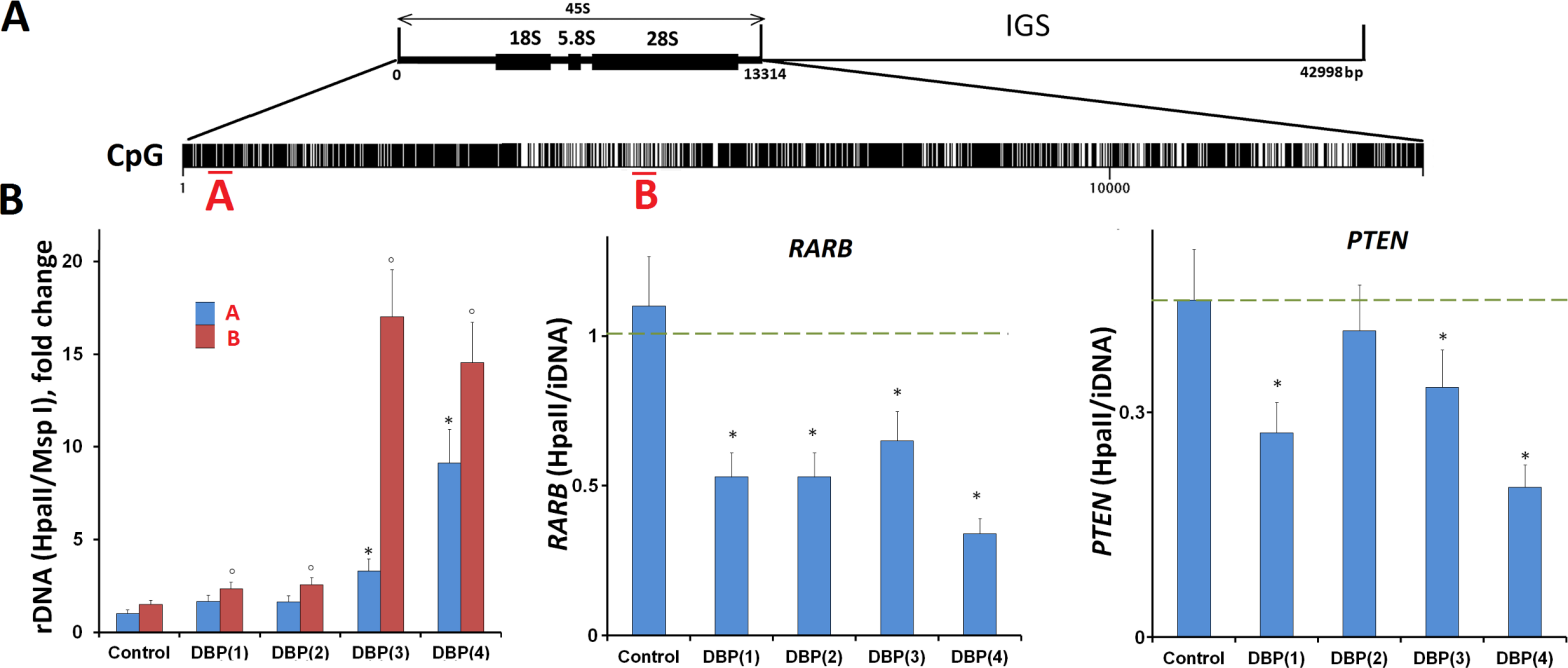
A. Distribution of CpG-motifs within rDNA (transcribed region of human ribosomal repeat). The digits indicate the nucleotide order number, the vertical bar shows the motif location. Red color is used to mark region A and region B, that were analyzed for the presence of methylated CCGG sites. B. Determination of methylation index of three genes in DNA from cells treated with 20 μM DBP(1–4), 72 h (description is given in Methods).

The 5-methylcytosine staining of the nuclei suggests, that the DBP(1–4) significantly increases ribosomal genes methylation together with the relative demethylation of the rest of the genome (Fig 7B). To confirm this hypothesis, the methylation of fragments A and B in the presence of DBP(1–4) was analyzed, Fig 8. QPCR analysis was performed for equal amounts of intact DNA (iDNA) samples, Msp1 and HpaII hydrolysates. During the hydrolysis of transcribed region of ribosomal repeat by methylation-insensitive endonuclease Msp1 some part of the rDNA always remains not hydrolyzed. Two reasons may explain that fact: a) the presence of strongly bound proteins in rDNA isolated from the cells that shield the restriction sites in a small number of rDNA copies and b) high rDNA polymorphism associated with increased frequency of mutations [15–17]. We found that in our experiments about 20 of 422 copies fragment A and about 10 copies fragment B detected by qPCR remain not hydrolyzed. The number of non-hydrolyzed copies in the control DNA and in DNA of DBP(1–4)-treated cells varied insignificantly (p > 0.05).

The methylation index for the rDNA equal to the ratio of the fragments A and B in HpaII and Msp1-hydrolysates increases in the cells cultivated with DBP(1–4) by 1.4–9.5 times for fragment A and by 1.5–15 times for fragment B (Fig 8B). The greatest effect was achieved in the presence of DBP (3, 4). Thus, the 5-methylcytosine localization in the nucleolus is confirmed by the increase of a fragment of transcribed region of ribosomal repeat methylation.

#### *RARB* and *PTEN* genes

Promoter regions of *RARB* and *PTEN* genes were selected as target fragments. *RARB* and *PTEN* promoter fragments contain one recognition site for restriction endonucleases HpaII/Msp1. In the DNA isolated from control MCF-7 culture, *RARB* genes were methylated in all the cells. It is shown by the fragment in the HpaII—hydrolysate and in the intact DNA (HpaII/iDNA) ratio equal to one (Fig 8D). For the *PTEN* gene the ratio is 0.45. Thus, in MCF-7 only about half of the cell culture contains 5-methylcytosine in *PTEN* gene analyzed site CCGG.

Fig 8D shows that in presence of DBP(1–4) the ratio (HpaII/iDNA) for the promoter region of the gene *RARB* reduced by 2–3 times respectively. For *PTEN* gene promoter region, the ratio of HpaII/iDNA was reduced significantly (1.3–2.2 fold) after treatment with DBP(1) and DBP(3,4) (Fig 8D).

### Reactivation of *CDKN2A*, *Apaf-1*, *RUNX3*, *APC*, *RARB* and *PTEN* by the DBP(1–4)

The genes described in the literature as transcriptionally silent due to the hypermethylation of the promoter regions were selected: *CDKN2A*, *Apaf-1*, *RUNX3*, *APC*, *RARB*, and *PTEN* [18–20] (Table 3). To evaluate an effect of the methylation inhibitors on the cells we studied the expression of particular genes in response to the introduction of these compounds. Relative levels of mRNA after the incubation of MCF7 cells with 20μM DBP(1–4) are presented in the Table 3. As control 2’-deoxy-5-azacytidine (AzaC) was added in cultivation medium for reactivation of genes by demethylation of their promoter regions.

**Table 3:** DBP(n) (20μM, 72h) effect on RNA level in MCF-7.

№	Gene	Control	AzaC	DBP			
				(1)	(2)	(3)	(4)
1	*RARB*	1.0 ± 0.2	3.3 ± 0.6*	2.7 ± 1.3*	2.2 ± 1.1*	1.4 ± 0.2	2.2 ± 0.4*
2	*PTEN*	1.0 ± 0.3	1.3 ± 0.3	4.7 ± 0.9*	4.2 ± 0.8*	3.1 ± 1.3*	2.5 ± 0.5*
3	*CDKN2A*	1.0 ± 0.1	1.3 ± 0.3	7.4 ± 1.5*	2.9 ± 0.5*	1.5 ± 0.3	1.4 ± 0.2
4	*Apaf-1*	1.0 ± 0.1	0.9 ± 0.1	2.3 ± 0.03*	1.9 ± 0.3*	1.4 ± 0.2	1.5 ± 0.2
5	*RUNX3*	1.0 ± 0.2	1.7 ± 0.4	2.7 ± 0.1*	2.6 ± 0.5*	1.2 ± 0.2	1.5 ± 0.3
6	*APC*	1.0 ± 0.2	0.7 ± 0.2	2.4 ± 0.05*	1.0 ± 0.2	0.8 ± 0.1	1.0 ± 0.2
7	*18S rRNA*	1.0 ± 0.1	-	0.67 ± 0.02*	-	-	0.76 ± 0.03*
8	5-ETS(1)	1.0 ± 0.2	-	0.25 ± 0.05*	-	-	0.23 ± 0.06*
9	5-ETS(2)	1.0 ± 0.1	-	0.38 ± 0.07*	-	-	0.35 ± 0.04*
10	3-ETS	1.0 ± 0.1	-	0.14 ± 0.03*	-	-	0.12 ± 0.02*

*) p < 0.05

The expression of all investigated genes significantly increased when the cells were incubated with the DBP(1). For most genes the expression increases more than after the incubation of the cells with AzaC. DBP(2) was able to increase the expression of all studied genes, except *APC*. For DBP(3) significant changes in gene expression were not observed, except *PTEN*. DBP(4) was able to increase the expression of *RARB* and *PTEN*.

### The influence of DBP(n) on the level of ribosomal RNA in MCF7

The increase of the 5-methylcytosine in the nucleolus (Fig 7) and the increase of the rDNA methylation (Fig 8) suggest that the amount of rRNA in the cells may be reduced. RT-qPCR was used to determine the amount of rRNA in the cells that have been cultured with the DBP(1) or DBP(4). These compounds differed by magnitude of rDNA demethylation effect significantly (Table 3, line 7). The 18S rRNA amount in the cells was reduced by 33% (DBP(1)) and 24% (DBP(4)).

Synthesis of RNA was estimated by measuring the change in numbers of 45S pre-rRNA fragments. Two 5'-ETS regions (5'-ETS core): 852–958 b (5'-ETS (1)) and 1285–1405 b (5'-ETS (2)) and a single 3'-ETS region (13117–13195 b) were analyzed. The cultivation of MCF7 cells in the presence of 20 μM DBP(1,4) for 72 hours reduced the number of 45S pre-rRNA fragments three fold for 5'-ETS (the values for 5'-ETS (1) and 5'-ETS (2) didn’t differ significantly from one another, p>0.05) and eight fold for 3'-ETS. In summary, the values for 5'-ETS (1,2) and 3'-ETS were significantly different (p < 0.05) compared to the control samples. Thus, the 18S rRNA level and 45S pre-rRNA level decreased during the treatment, but the changes did not correlate with the magnitude of the methylation effect.

### DiscussionDBP(1–4) distribution in MCF-7 cells

**Fig 9:**
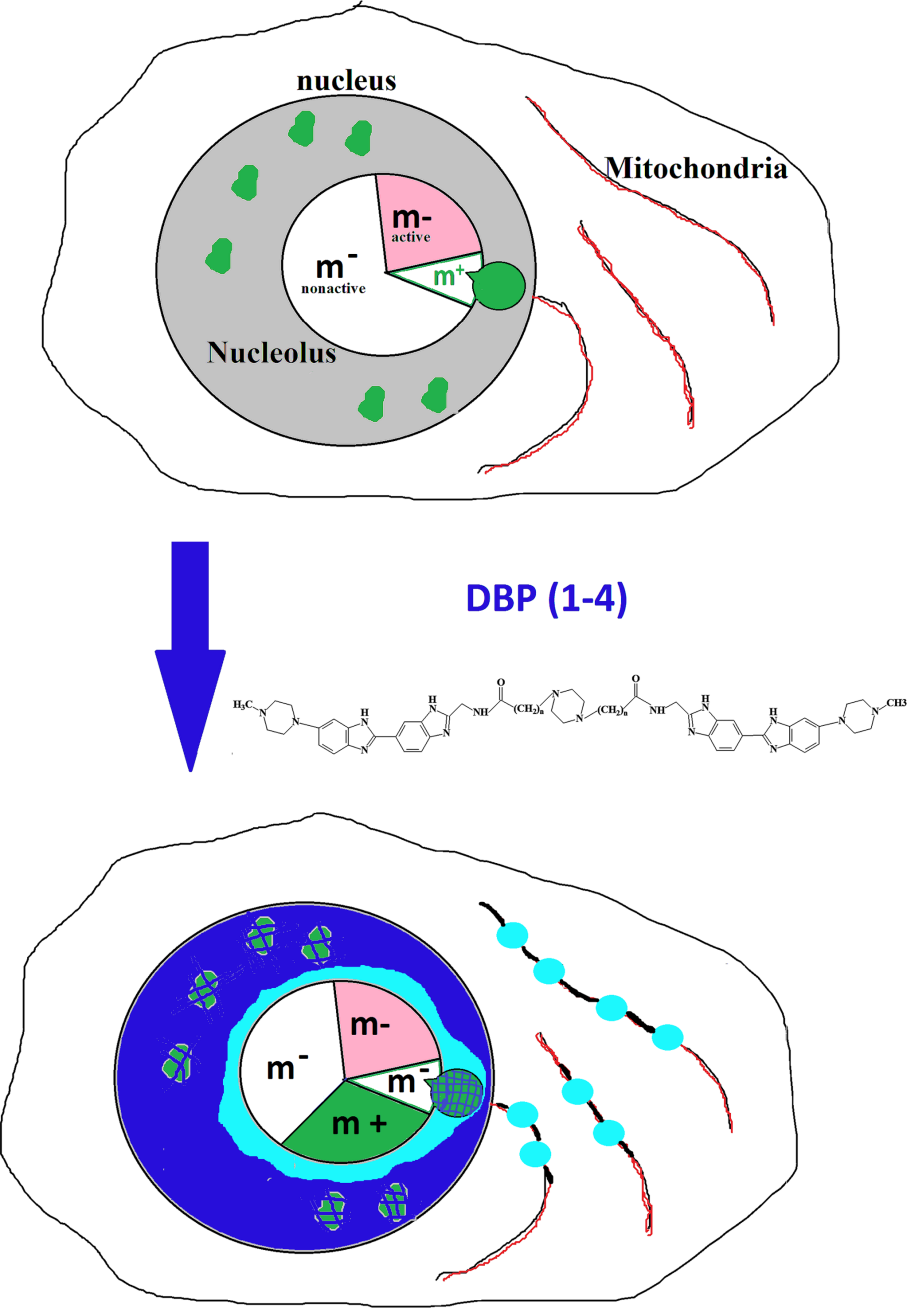
Distribution of DBP(n) in MCF-7 and the change of DNA methylation in the nucleus. Intact cells contain hypermethylated DNA loci in the nucleus (green granules). DBP(n) penetrates into the cells and is localized in the mitochondria, in the nucleus and in the areas near nucleolus. In the nucleolus the amount of DBP(n) is much lower than in other parts of the nucleus. Light blue color (washed in the presence of detergent)- DBP(n) interacts with proteins. Dark blue color (resistant to treatment with detergent)—DBP(n) interacts with nuclear DNA blocking the MTase activity. The number of hypermethylated loci in the nucleus after DBP(n) treatment is significantly reduced. MTase molecules from the nucleus migrate to the nucleolus where they methylate rDNA. As a result nuclear sequences outside the nucleolus are demethylated, while rDNA methylation in the nucleolus increases. On figure of nucleolus by a circular histogram we show actively transcribed rDNA copies (pink color), not transcribed and nonmethylated rDNA (white color) and hypermethylated rDNA (green). We assume that predominantly increases the methylation of transcriptionally inactive rDNA copies.

Fig 9 summarizes the obtained data: the DBP(1–4) penetrates living cells and accumulates primarily in the mitochondria of MCF-7. The accumulation takes place not along the entire length of the mitochondria, but in some areas only. In the place of the DBP(1–4) accumulation in the mitochondria the mitochondrial potential decreases, as shown by the decrease of TMRM MitoTracker fluorescence intensity. The change of mitochondria potential is not accompanied by the increased ROS levels in the cells when DBP(1–4) are used in concentrations not exceeding 20 μm. Apparently, the DBP(1–4) in mitochondria interact not with the DNA, but with mitochondrial proteins. Two facts prove this hypothesis: 1) the change of the DBP(1–4) fluorescence wavelength: bright blue fluorescence is observed in contrast to dark blue typical for the DBP complexes with the DNA; 2) the signal is not detected in the mitochondria of fixed cells treated with a detergent, which removes some cellular proteins.

At least two subpopulations of MCF-7 cells are detected with cell staining and TMRM fluorescence analysis. Apparently, a part of the cells either quickly eliminates the DBP(1–4) from the cytoplasm or has low membrane permeability for DBP(1–4). The penetration and accumulation of significant quantities of the DBP(1–4) in the nucleus correlates with a significant fluorescence quenching of Mitotracker.

The nuclei staining in living MCF-7 cultured in the presence of the DBP(1–4) is heterogeneous. Dark blue color of the nuclei indicates nuclear DNA/DBP(1–4) complexes [10]. The nucleoli in the nuclei of the cells are strongly contrasted. The fluorescence parameters of the nucleolus in the presence of the DBP(1–4) are similar to the parameters of the DBP(1–4) fluorescence in the mitochondria of MCF-7: a bright light blue fluorescence with the signal disappearing after the treatment of fixed cells with the detergent. We propose that the DBP (1–4) interacts not with the DNA, but with the proteins on the surface of the nucleolus. A few molecules of the DBP (1–4) penetrate the nucleolus, as evidenced by the absence of staining of the nucleoli in fixed cells. It may be explained by high amount of GC-pairs in ribosomal genes (see Fig 8C). It is known that bisbenzimidazole-containing compounds interact primarily with AT-rich sequences of the genome [10]. Thus, the DBP(1–4) are concentrated in close proximity to the nucleolus, while inside the nucleolus the concentration of these compounds is reduced.

It was found previously that the DBP is low-toxic for normal cells [10]. In our study we found that the DBP (1–4) are practically non-toxic at concentrations not exceeding 20 μM in the culture medium. DBP(2) stimulates the proliferation, and DBP(4) slightly reduces it. However, the number of proliferating cells and the number of the cells in different phases of the cell cycle does not change significantly. Absence of high toxicity is important in the study of the effects of these compounds on genome methylation. DNMT1 MTase methylates DNA after cell replication, therefore to obtain clones with demethylated DNA, the cells have to divide.

### The DBP(1–4) change the methylation profile of the MCF-7 genome

The DBP(1–4) exert relatively little effect on the total genomic DNA methylation. The most profound demethylation was observed after treatment with DBP(1) that was found to significantly reduce the 5-methylcytosine level in DNA by about 40%. The DBP(2–4) had little or no effect on the 5-methylcytosine level in the DNA.

We hypothesized that the demethylation is limited to a few genes only, so it is impossible to estimate the content in the DNA by a general analysis of 5-methylcytosine. Therefore, we studied the reactivation of several genes that are not expressed in MCF-7 due to the methylation of their promoters [18–20]: *CDKN2A*, *Apaf-1*, *RUNX3*, *APC*, *RARB*, and *PTEN*. DBP (1–4) induce a significant increase of the expression of all these genes. It is worth to mention that the effect of DBP(1–4) for most genes was more pronounced than for the standard demethylation agent AzaC. The greatest effect was observed for DBP(1) and DBP(2).

For *RARB* and *PTEN* genes, it has been shown that a significant increase in gene expression correlates with the demethylation of their promoters in DNA from cells treated with the DBP(1–4). Thus, we conclude that all DBP(1–4) and not only DBP(1) demonstrate the ability to block the MTase activity.

A weak total demethylation effect found by 5-methylcytosine may be explained by a significant 5-methylcytosine redistribution in the various genome sequences. In addition, the population of MCF-7 may contain various clones that can be methylated differently. Thus, exploring the effects of DBP(n) compounds on genome methylation it is necessary to conduct a whole-cell analysis of the 5-methylcytosine distribution in the cells of the culture. To understand why the demethylation effect is not evident by the level of total 5-methylcytosine, we used a fluorescence microscopy to study the localization of methylated DNA in the cells.

In control cells the areas with increased DNA methylation were observed in agreement with the literature about the hypermethylation of MCF-7 cells genome sequences. The staining observed was absolutely identical to the data published previously [21]. The DBP(1–4) significantly alter the methylation pattern of DNA sequences in the cell nucleus. The predominant number of 5-methylcytosine in the presence of the DBP (1–4) is localized in the nucleoli of the cells. The amount of the 5-methylcytosine in the other regions of the nuclei is significantly lower. Moreover, contrasting nucleolus was not observed in control cells. According to the data in intact MCF-7 cells rDNA methylation is rather low—less than 15% of 422 rDNA copies contain methylated CCGG binding sites in the region A.

The transcribed region of ribosomal repeat contains a very large number of CpG sites (Fig 8C) that can potentially be methylated. The data obtained using HpaII/Msp1 confirm the assumption that the rDNA methylation increases in the cells treated with DBP (1–4). The methylation of the genes located outside the nucleolus (*RARB* and *PTEN*) is reduced. Most probably this interesting fact is explained by binding of the DBP (1–4) with the DNA that blocks the MTase Dnmt1 responsible for maintaining the DNA methylation in the nucleus. Therefore, in the genes located in the nucleus and producing complexes with DBP(1–4) the DNA demethylation was observed. The DBP(1–4) do not penetrate in the nucleolus accumulating on its surface. Here the DBP interact with the proteins and with AT-rich satellite heterochromatin sequences surrounding the nucleolus [22]. In the same heterochromatin area outside the nucleolus hypermethylated rDNA copies are usually localized [23]. The MTases which cannot effectively bind to the DNA complexes with DBP (1–4) in the nucleus migrate to the nucleolus. Since the DBP (1–4) concentration in the nucleolus is low, MTase may interact with the rDNA and methylate numerous CpG sites in the transcribed region of the rDNA copies.

The nucleolus sequesters a large number of proteins that temporarily are not needed for the cell, e.g. stress proteins. Under the stress these proteins migrate from the nucleolus to participate in the stress response [24]. Also the nucleolus may sequester MTases that cannot effectively bind to other sequences in the nucleus due to the DNA-DBP (1–4) complexes.

The characteristics of MCF-7 cells observed in this study differ from the data previously obtained for HeLa line of cancer cells. Exploring the DB(n) methylation effects of the rDNA in HeLa cells by methylation-sensitive endonucleases a decrease of rDNA methylation was found [9]. That contradiction may be explained by two facts. First, HeLa cells contain much more hypermethylated rDNA copies than MCF-7 [25]. These copies are located outside the nucleolus. In HeLa nuclei the nucleoli are localized in close proximity to large blocks of hypermethylated DNA [25], which also seem to include hypermethylated rDNA copies. In the presence of DB(n) maintaining the rDNA methylation outside the nucleolus does not occur and the total rDNA methylation may be reduced. Second, possibly in contrast to the DBP(1–4) the DB(n) penetrate into the nucleolus to participate in rDNA demethylation.

The increase of rDNA methylation level in the presence of DBP (1–4) three days after the start of cultivation does not change significantly the rRNA amount in the cells. The 18S rRNA amount is reduced less than 30% and does not depend on the level of additional rDNA

methylation in the presence of the DBP. However, three days after cultivation significant changes were observed in transcription and/or processing of the 45S pre-rRNA (Table 3). The number of 5'-ETS (1,2) fragments decreased by 3–4 times compared to the control. The number of 3'-ETS fragments decreased by 7–8 times. It is assumed that the decrease in 3'-ETS reflects the number of whole 45S pre-rRNA molecules, since the 3'ETS fragment degrades very rapidly and is not detected in free form outside of the 45S pre-rRNA molecule [26]. The 5'-ETS (1,2) fragments degrade slowly and therefore may be detected in the composition of pre-rRNA fragments that are produced during processing of the full-sized 45S pre-rRNA transcript. These results may be explained first by a significant reduction of ribosomal RNA gene transcription, and second, by decrease of the 5'-ETS processing speed. As a result, an accumulation of 5'-ETS (1,2) fragments compared to 3'-ETS fragment occurs.

It is known that ribosomal genes may be divided into transcription-active (approximately 30% of rDNA copies) and silent [27]. Transcription-active copies are demethylated. Inactive copies are divided into two fractions: demethylated (or hypomethylated) and hypermethylated (Fig 9). It is assumed that rDNA copies temporarily not needed for the cell’s protein synthesis are sequestered in the nucleolus interacting with the transcriptional inactive rDNA copies. Apparently, it is transcriptionally inactive hypomethylated rDNA copies that primarily bind excessive MTase molecules and are exposed to additional methylation during treatment with DBP (1–4), which blocks enzyme interaction with the DNA sequences outside the nucleolus (Fig 9). Transcription-active copies may be protected from the MTases by Pol I and its accessory proteins that perform transcription. However, it is known that the inactive demethylated copies participate in transcription by influencing the nucleolus chromatin structure. As a result of the inactive rDNA copies, methylation of the active rDNA copies may be reduced. Meanwhile, we cannot exclude that the prolonged DBP (1–4) effect may contribute to the methylation of transcriptionally active rDNA copies. Potentially this effect may lead to cessation of proliferation and death of cancer cells.

## Conclusions

Three potentially useful properties of the dimeric bisbenzimidazoles that contain a piperazine cycle in the linker are described. (1) the DBP (1–4) can accumulate in the mitochondria of cancer cells, reducing mitochondrial potential; (2) the DBP (1–4) cause the demethylation of the genome of cancer cells, including demethylation of promoters of tumor suppressor genes; (3) the DBP (1–4) significantly increase the methylation of ribosomal genes in the nucleoli of cells. Further study of the properties of these compounds can lead to new anticancer agents.

## Abbreviations

DB(n): dimeric bisbenzimidazoles containing oligomethylene linkers between bisbenzimidazole fragments
DBP(n): dimeric bisbenzimidazole containing a central 1,4-piperazine residue in oligomethylene linkers
5mC: 5-methyl-2’-deoxycytidine
MTase: C5-cytosine-DNA-methyltransferase
TMRM: tetramethylrhodamine methyl ether
FCA: flow cytometry analysis
DCF: 2’,7’- dichlorofluorescein
DCHF- DA: 2’,7’-dichloro-dihydro-fluorescein diacetate
ROS: reactive oxygen species
